# Supplementary material for: The metabolic profile of a rat model of chronic kidney disease
Source: PeerJ. 2017 May 23;5:e3352. doi: 10.7717/peerj.3352 (PMC5444364; doi:10.7717/peerj.3352)
Supplement: Supplemental Information 1 [file peerj-05-3352-s001.docx]

**Supplementary Material**

**A metabolic profile of a rat model of chronic kidney disease**

Yohei Tanada^1#,^ Junji Okuda^1#^, Takao Kato^1*^, Eri Minamino-Muta^1^, Ichijiro Murata^2^, Tomoyoshi Soga^3^, Tetsuo Shioi^1^, Takeshi Kimura^1^

1. Department of Cardiovascular Medicine, Graduate School of Medicine, Kyoto University, 54 Kawahara-cho, Sakyou-ku, Kyoto City, Kyoto, Japan

2.Department of Nephrology, Gifu University Graduate School of Medicine, 1-1 Yanagido, Gifu City, Gifu, Japan

3. Institute for Advanced Biosciences, Keio University, Kakuganji Mizukami 246-2, Tsuruoka City, Yamagata, Japan

# These authors contributed equally to this work

**Supplementary Table 1.** Primer sequences used in real time quantitative RT-PCR

| Gene | Forward | Reverse | GenBank Entry |
| --- | --- | --- | --- |
| 18SrRNA | AGTCCCTGCCCTTTGTACACA | CGATCCGAGGGCCTCACTA | M11188 |
| Glut 1 | CCCCAGAAGGTAATTGAGGA | GATTAACAAAGAGGCCCACA | NM_138827.1 |
| Gllut 4 | TGGCATGGGTTTCCAGTATG | GGTTTCACCTCCTGCTCTAA | NM_012751 |
| HK, type 1 | AGGAAGGAGACCAACAGCAA | TTCCCACTGCGGATCTTTAC | NM_012734 |
| HK, type 2 | CACTCCAGATGGCACAGAGA | TAACCTCCGTGGGATGGAG | NM_012735 |
| PFK 1 | GTGGAAACCCAACTCCCTTT | CCCCAGAACACAACCTGAGT | NM_031715 |
| GAPDH | GGATGCAGGGATGATGTTCT | GAAGGGCTCATTGACCACAGTT | NM_017008.3 |
| PGAM2 | TGGCTGGTTTGATGCAGA | ACCCACATTTGGTCCGTA | NM_017328.1 |
| TIGAR | CGGAATTCAGAACAGTTTTCCCAAGGATCTCC | GGAATTCAACCTTAGCGAGTTTCAGTCAGTCC | NP_001020235.1 |
| HIF-1α | TGCTTGGTGCTGATTTGTGAA | TATCGAGGCTGTGTCGACTGAG | AF_057308 |
| ACLY | GGCAAGATCCTCATCATTGGA | CAACTTCTCCCATCACTCGTA | BC100618.1 |
| ACS | AACACGTCAGTGAAGCGATG | AACACATTTGCCCCTTTCAC | NM_012820.1 |
| CPT 1 | ATGTTTGACCCAAAGCAGTA | TCATGTAGGAAACCCCGTAT | BC085761.1 |
| ACC a | ATGATTGCTGGGGAATCCTCA | GAGGTGTATACTTCCCGACCA | Rn.44372 |
| ACC b | GCTGTGTGAGAGATGTGGA | GCACCTGTCTGAAAAAGCA | NM_053922 |
| FAT/CD36 | GACCATCGGCGATGAGAAA | CCAGGCCCAGGAGCTTTATT | AK_072411 |
| VLCAD | CTCCTCTGATGCTTCCACCA | GTCCCACCAGCTCTTTGAGA | NM_012891 |
| LCAD | TTTTCCGGGAGAGTGTAAGGA | CTCTGCAATGTTGATGCCAA | NM_012819 |
| MCAD | GGGACTAGGGTTTAGCTTCGA | CCCAGGCTCTCTTGATGAGA | NM_016986 |
| ABCA1 | ATGCACCAGAGCGCGAGCTG | CTCCTGCCGCATGTCGCTCC | NM_178095.2 |
| SREBF 1 | TCACTGAAAGACCTGGTGTCA | GCTTTCACCTGGTTATCCTCA | AF286470.2 |
| SREBF 2 | CAAGTACCTGCAGCAGGTCA | AGTCAATGGAATAGGGGGAGA | NM_001033694 |
| PGC1α | CACCAAACCCACAGAGAACAG | GCAGTTCCAGAGAGTTCCACA | NM_031347 |
| PPARα | ACCCGAGAGAGTTCCTAAAGAA | AATGTCACTGTCATCCAGTT | NM_013196.1 |
| NRF 1 | TTACTCTGCTGTGGCTGATGG | CCTCTGATGCTTGCGTCGTCT | NM_10938 |
| NRF 2 | CACCACACTCAACATTTCGG | CCTTGGGGACCTTTGAACTT | XM_344002 |
| TFAM | GAAAGCACAAATCAAGAGGAC | CTGCTTTTCATCATGAGACAG | NM_031326 |
| UCP3 | GCCGCTTGGTACTTTCCTCAT | CGACCCCTGGGCAGAGA | AF_035943 |
| ANT | TGCTCAAGTTCACAGGTTCAC | CTTCTGTTTGCTGTGGAATC | [NP_599172.1](javascript:PopUpMenu2_Set(Menu_prot77736542);) |
| SDHb | TGGCTTTCACTTCTCTGTTGG | ATCTCCAGTTGTCCTCTTCCA | NM_001100539.1 |
| αS9 | CTGATGCCATCTTCATACGA | CGTTAGCAATCCCTTTGGAA | BC168777.1 |
| ND4 | CCTACCCTCAACATGATCCAA | CATAACCCCCTAGCTTTAGGA | RGD:620559 |
| Cyt-b | TCTCATCAGTCACCCACATC | CATTCTGGTTTGATGTGGGG | BC081820. |
| Cyt-c | ATGGGTGATGTTGAAAAAGG | TTATTCATTAGTAGCCTTTT | NC_005103.4 |
| COX-1 | AGCAGGAATAGTAGGGACAGC | TGAGAGAAGTAGTAGGAGGCC | X14848 |
| COX-4 | TGGGAGTGTTGTGAAGAGTGA | GCAGTGAAGCCGATGAAGAAC | X15029 |
| COX-5a | CTGCCGCTGTCTGTTCCATTCG | TGTCACCCAGCGAGCATCAAACT | NM_145783.1 |
| CS | TGTGGACATGATGTACGGTGG | ACTATAGCCTCGGAAGCGGA | NM_130755.1 |
| ACO2 | CGAGCAGACATTGCCAACCTA | GTGAGCCAAGTCAGGGGTAAA | NM_024398.2 |
| OGDH | TCCGGAAGCCGTTAATCGT | TCTTCTGGGATCACACGCTG | NM_001017461.1 |
| SUCLG1 | GGAGCGACAGCTTCTGTCAT | GTCCTGCTGCGGAATACCTT | NM_053752.2 |
| FH | GAGGTGCGACGGAGAGAATG | CCTTCAGCTACCTCATCTGCG | NM_017005.2 |
| MDH2 | ATGTGAAAGGCTACCTCGGG | AGCATTGGTGTTGAACAGGTC | NM_031151.2 |
| NPHS1 | ACACCAACATCCAGCTCGTC | GGGCCTCGTACCTGATTTGGA | NM_022628.1 |
| NPHS2 | ATTCCGACTGGGACATCTGC | GGTTACCACCTCATGGAAAGGT | BC098649.1 |
| KIM1 | GTGGTTGTCACCAGGTACATCAT | GTTGTCTTCAGCTCGGGGAT | NM_173149.2 |
| NGAL | CAACGTCACTTCCATCCTCGT | CTGTATCTGAGGGTAGCTGTGAAT | NM_130741.1 |
| Osteopontin | CCATGCAGAGAGCGAGGATT | GTCAGGGACATCGACTGTGG | NM_012881.2 |
| Collagen1 | GAATATGTATCACCAGACGCAGAAG | TAGGACATCTGGGAAGCAAAGTT | NM_053304.1 |
| αSMA | GGCCAACCGGGAGAAAATGA | GTCCAGCACAATACCAGTTGT | NM_031004.2 |

18SrRNA; 18S ribosomal RNA, Glut; Glucose transporter, HK; hexokinase, PFK; phosphofructokinase, GAPDH; glyceraldehyde-3-phosphate dehydrogenase, PGAM; phosphoglycerate mutase, TIGER; TP53 induced glycolysis regulatory phosphatase, HIF-1α; Hypoxia Inducible Factor 1-α, ACLY; ATP citrate lyase, ACS; Acyl-CoA synthetase, CPT-1; Carnitine palmitoyltransferase-1, ACC; Acetyl-CoA Carboxylase, FAT/CD36; fatty acid translocase / cluster of differentiation 36, VLCAD; very-long-chain acyl-CoA dehydrogenase, LCAD; long-chain acyl-CoA dehydrogenase; MCAD;medium-chain acyl-CoA dehydrogenase, ABCA1; ATP-Binding Cassette 1, SREBF; sterol regulatory element binding transcription factor; PGC1-α; peroxisome proliferator-activated receptor γ coactivator1-α, PPARα; peroxisome proliferator-activated receptor α, NRF; nuclear respiratory factor, TFAM; mitochondrial transcription factor A, UCP3; uncoupling protein 3, ANT; adenine nucleotide translocator, SDHB; succinate dehydrogenase b, αS9; alpha-subcomplex 9, ND4; NADH dehydrogenase 4, Cyt; cytochrome, COX; cytochrome c oxidase, CS; citrate synthase, ACO2; aconitase 2, OGDH; oxoglutarate dehydrogenase, SUCLG1; succinate-CoA Ligase-1, FH; fumarate hydratase, MDH2; Malate Dehydrogenase 2, NPHS; nephrosis, KIM1; kidney injury molecule-1, NGAL; neutrophil gelatinase-associated lipocalin, αSMA; α-smooth muscle actin.

**Supplementary Table 2.** **Concentration of metabolites of kidney identified by metabolome analysis**

|  | **11 week** | | | | | | **21 week** | | | | | |
| --- | --- | --- | --- | --- | --- | --- | --- | --- | --- | --- | --- | --- |
|  | **Low-Salt (mmol/g)** | | | **High-Salt (mmol/g)** | | | **Low-Salt (mmol/g)** | | | **High-Salt (mmol/g)** | | |
| Numbers of Animals | 8 | | | 12 | | | 8 | | | 11 | | |
| G6P | 10.11 | ± | 0.98 | 15.31 | ± | 4.57 | 11.18 | ± | 0.84 | 11.16 | ± | 0.74 |
| G1P | 24.38 | ± | 1.44 | 23.75 | ± | 1.56 | 21.88 | ± | 1.39 | 15.87 | ± | 1.21^†^ |
| F6P | 7.03 | ± | 1.14 | 7.84 | ± | 0.98 | 8.68 | ± | 1.26 | 6.21 | ± | 0.40 |
| F1,6BP | 9.81 | ± | 2.47 | 12.50 | ± | 3.16 | 17.24 | ± | 3.08 | 14.89 | ± | 1.94 |
| 3PG | 44.38 | ± | 3.60 | 41.25 | ± | 3.52 | 43.50 | ± | 3.33 | 48.55 | ± | 3.38 |
| 2,3-DPG | 7.33 | ± | 1.67 | 7.39 | ± | 1.05 | 8.74 | ± | 1.45 | 4.04 | ± | 0.46^†^ |
| 2PG | 7.56 | ± | 0.47 | 7.25 | ± | 0.57 | 6.63 | ± | 0.56 | 7.09 | ± | 0.68 |
| PEP | 14.75 | ± | 1.11 | 12.42 | ± | 0.97 | 12.66 | ± | 1.18 | 8.93 | ± | 0.72^†^ |
| Acetyl-CoA | 2.96 | ± | 0.32 | 3.48 | ± | 0.34 | 3.95 | ± | 0.53 | 1.68 | ± | 0.11^†^ |
| Citrate | 58.38 | ± | 9.98 | 59.42 | ± | 9.24 | 78.25 | ± | 6.71 | 320.45 | ± | 39.45^†^ |
| Cis-Acotinate | 1.47 | ± | 0.26 | 2.69 | ± | 0.63 | 2.90 | ± | 0.60 | 23.11 | ± | 3.03^†^ |
| Isocitrate | 3.84 | ± | 0.40 | 3.64 | ± | 0.32 | 3.50 | ± | 0.19 | 12.51 | ± | 1.26^†^ |
| Succinate | 637.13 | ± | 31.07 | 624.08 | ± | 35.87 | 674.50 | ± | 40.05 | 407.64 | ± | 41.59^†^ |
| Fumarate | 153.88 | ± | 13.52 | 170.08 | ± | 10.49 | 126.50 | ± | 14.72 | 148.45 | ± | 14.44 |
| Marate | 380.88 | ± | 24.20 | 409.67 | ± | 20.17 | 322.38 | ± | 18.03 | 364.82 | ± | 29.69 |
| Lactate | 3368.88 | ± | 141.78 | 3631.67 | ± | 133.85 | 3161.88 | ± | 188.00 | 4048.73 | ± | 278.47^†^ |
| Malonate | 5.36 | ± | 0.20 | 5.06 | ± | 0.43 | 5.18 | ± | 0.47 | 3.78 | ± | 0.39^†^ |
| 3-Hydroxybutyrate | 117.25 | ± | 5.94 | 118.67 | ± | 7.29 | 113.00 | ± | 6.47 | 89.45 | ± | 8.97^†^ |
| 2-Hydroxyglutarate | 8.23 | ± | 0.37 | 10.37 | ± | 0.80 | 9.13 | ± | 0.81 | 7.69 | ± | 0.73 |
| Pyridoxamine 5-P | 1.38 | ± | 0.26 | 1.47 | ± | 0.20 | 1.31 | ± | 0.26 | 0.72 | ± | 0.05^†^ |
| Serine | 804.25 | ± | 36.89 | 918.00 | ± | 45.97 | 584.00 | ± | 35.09 | 832.00 | ± | 61.79^†^ |
| Glycine | 3852.38 | ± | 170.53 | 3823.08 | ± | 171.77 | 3287.50 | ± | 197.31 | 3314.64 | ± | 266.40 |
| Betaine | 908.50 | ± | 45.39 | 1246.75 | ± | 83.49^*^ | 744.50 | ± | 59.83 | 1101.36 | ± | 159.19^†^ |
| Glutathione (ox) | 37.14 | ± | 17.43 | 35.69 | ± | 22.07 | 89.78 | ± | 26.64 | 112.74 | ± | 31.60 |
| Glutathione (red) | N.D. | | | N.D. | | | N.D. | | | N.D. | | |
| Taurine | 4122.88 | ± | 208.35 | 4192.33 | ± | 128.37 | 4484.88 | ± | 204.98 | 3903.09 | ± | 191.38^†^ |
| Isethionate | 5.05 | ± | 0.35 | 5.10 | ± | 0.45 | 7.05 | ± | 0.65 | 6.92 | ± | 0.87 |
| Alanine | 1037.13 | ± | 51.44 | 1176.17 | ± | 49.54 | 792.88 | ± | 54.91 | 1162.27 | ± | 91.52^†^ |
| Β-Alanine | 69.75 | ± | 5.41 | 59.42 | ± | 3.16 | 65.00 | ± | 5.27 | 44.73 | ± | 3.69^†^ |
| Aspartate | 1159.75 | ± | 57.72 | 1250.75 | ± | 71.74 | 917.63 | ± | 58.32 | 951.82 | ± | 59.06 |
| Carnosine | 3.43 | ± | 0.38 | 16.52 | ± | 13.59 | 1.85 | ± | 0.09 | 1.05 | ± | 0.15 |
| Histamine | 4.25 | ± | 1.56 | 1.44 | ± | 0.39 | 3.38 | ± | 1.43 | 5.46 | ± | 3.00 |
| Anserin | 4.19 | ± | 0.41 | 19.25 | ± | 15.38 | 5.56 | ± | 0.60 | 3.71 | ± | 0.72 |
| Histidine | 175.50 | ± | 10.37 | 194.00 | ± | 7.50 | 155.00 | ± | 12.38 | 189.27 | ± | 14.01^†^ |
| Glutamate | 7286.75 | ± | 271.53 | 7842.25 | ± | 234.88 | 6669.00 | ± | 288.77 | 5695.09 | ± | 430.52^†^ |
| Citrulline | 28.13 | ± | 1.77 | 27.83 | ± | 1.53 | 26.75 | ± | 1.50 | 49.45 | ± | 5.22^†^ |
| Arginine | 289.88 | ± | 17.29 | 317.75 | ± | 15.73 | 231.88 | ± | 16.12 | 333.45 | ± | 21.30^†^ |
| Ornithine | 63.50 | ± | 2.66 | 53.42 | ± | 1.85 | 47.88 | ± | 2.28 | 51.27 | ± | 3.98 |
| Proline | 340.00 | ± | 16.65 | 372.25 | ± | 17.66 | 237.25 | ± | 13.70 | 338.45 | ± | 26.06^†^ |
| Hydroxyproline | 119.00 | ± | 7.36 | 95.08 | ± | 5.58 | 54.25 | ± | 3.17 | 65.00 | ± | 6.49 |
| Creatine | 2090.50 | ± | 240.71 | 2223.58 | ± | 188.38 | 2607.38 | ± | 212.54 | 1663.73 | ± | 153.99^†^ |
| Creatinine | 45.50 | ± | 3.28 | 45.08 | ± | 2.36 | 67.13 | ± | 6.19 | 112.00 | ± | 8.36^†^ |
| Urea | 9716.3 | ± | 788.8 | 13450.6 | ± | 808.8 | 10193.0 | ± | 689.0 | 16455.8 | ± | 2829.9^†^ |
| Putrescine | 3.21 | ± | 0.11 | 3.40 | ± | 0.18 | 2.99 | ± | 0.25 | 4.17 | ± | 0.35^†^ |
| Spermidine | 15.35 | ± | 1.35 | 17.08 | ± | 1.04 | 12.06 | ± | 1.00 | 10.93 | ± | 1.09 |
| Spermine | 0.87 | ± | 0.07 | 0.81 | ± | 0.07 | 0.87 | ± | 0.12 | 0.81 | ± | 0.12 |

G6P; glucose-6-phosphate, G1P; glucose-1-phosphate, F6P; fructose-6-phosphate, F1,6BP; fructose-1,6-bisphosphate, 3PG; 3-Phosphoglycerate, 2,3-DPG; 2,3-diphosphoglycerate, 2PG; 2-phosphoglycerate, PEP; phosphoenolpyruvate, N.D.; not determined. Values are the mean ± SEM. *p<0.05 versus Low-Salt 11 week. †p<0.05 versus Low-Salt 21 week.

**Supplementary Figure Legend**

**Supplementary Figure 1.** (A) Hemodynamic parameters in DS rats fed a high-salt (HS) or low-salt (LS) diet. SBP: systolic blood pressure. (B) Echocardiographic data in DS rats fed an HS diet and an LS diet. IVSd: interventricular septum dimension; LVDd: left ventricular diastolic dimension; LVDs: left ventricular systolic dimension; FS: fractional shortening. Values are the mean ± SEM. (C) The blood urea nitrogen (BUN) and creatinine (Cre) levels in rats fed an HS diet and an LS diet. LS-11week, n=8; LS-21week, n=12; HS-11week, n=8; HS-21week, n=11. Values are the mean ± SEM. *p<0.05 versus LS-C group; ♰p<0.05 versus rats at 11 weeks of age.

**Supplementary Figure 2.** (A) Representative images of Sirius Red staining and (B) the area of interstitial fibrosis. At 21 weeks of age, the area of fibrosis in the kidney significantly increased in the HS group compared to that in the LS group. There was no difference between the HS and LS groups at 11 weeks of age. LS-11week, n=8; LS-21week, n=12; HS-11week, n=8; HS-21week, n=11. The bars represent 50 µm. Values are the mean ± SEM. *p<0.05 versus LS-C group. Original magnification is x200.

**Supplementary Figure 3.** (A) The amounts of NAD and NADH were measured in metabolome analysis. In the HS group at 21 weeks of age, the levels of NAD+ and NADPH decreased. LS-11week, n=8; LS-21week, n=12; HS-11week, n=8; HS-21week, n=11. Values are the mean ± SEM. *p<0.05 versus LS-C group; ♰p<0.05 versus rats at 11 weeks of age. (B) Protein expression of NDUFA9 in western blotting at 21 weeks of age. L: LS-21week; H: HS-21week. LS-21week, n=7; HS-21week, n=7. Values are the mean ± SEM.

**Supplementary Figure 1**

**
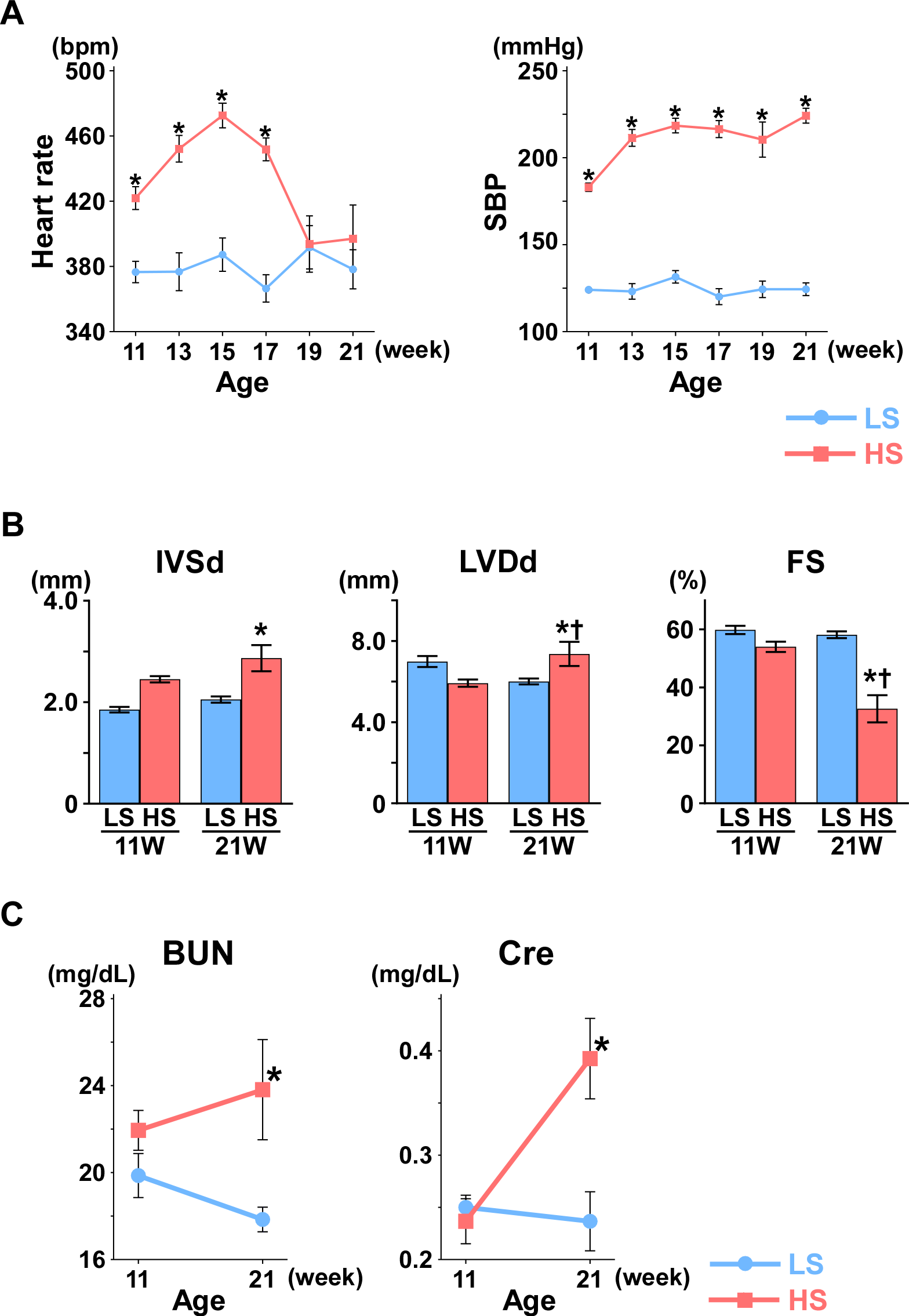
**

**Supplementary Figure 2**

**
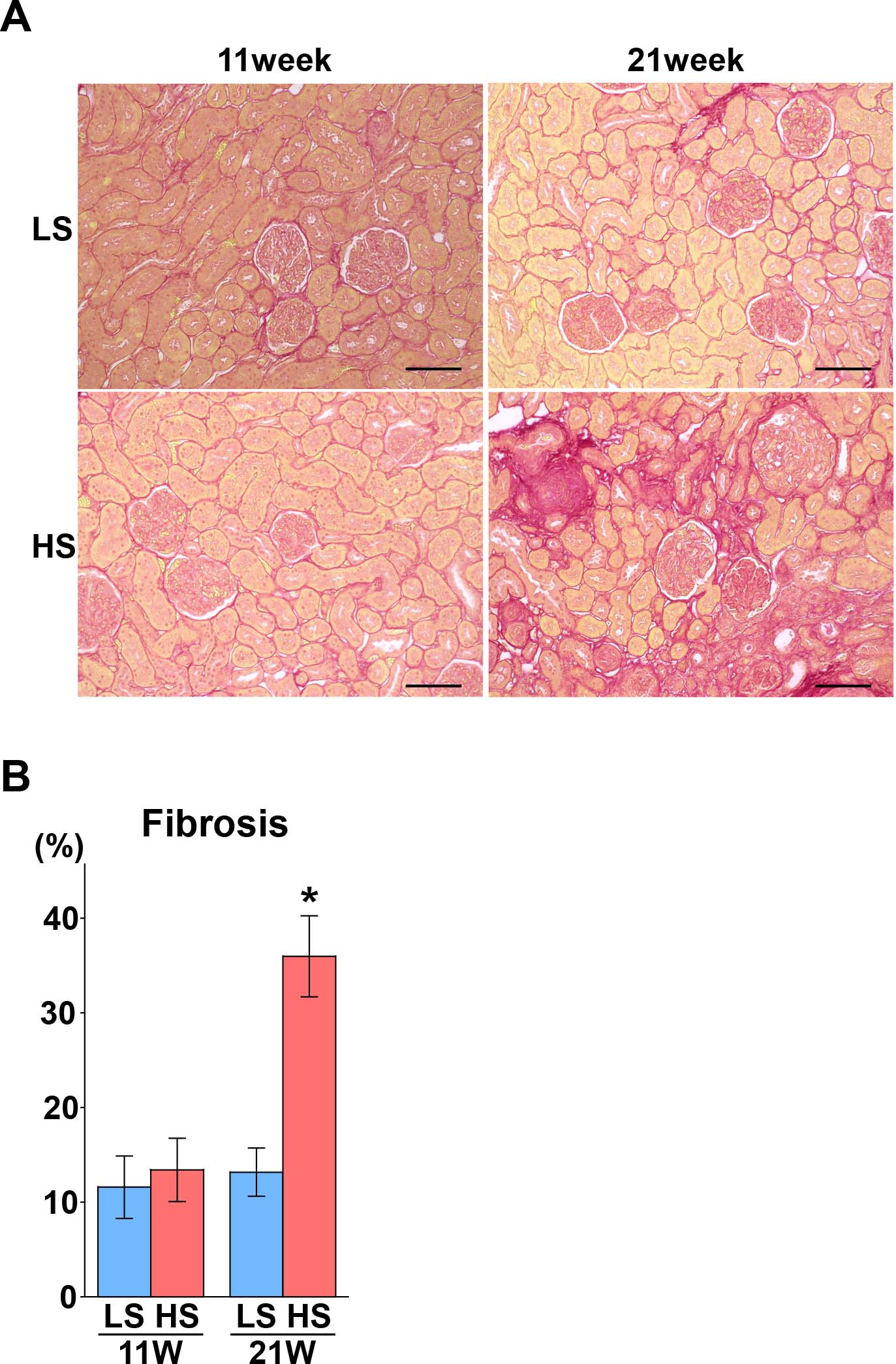
**

**Supplementary Figure 3**

**
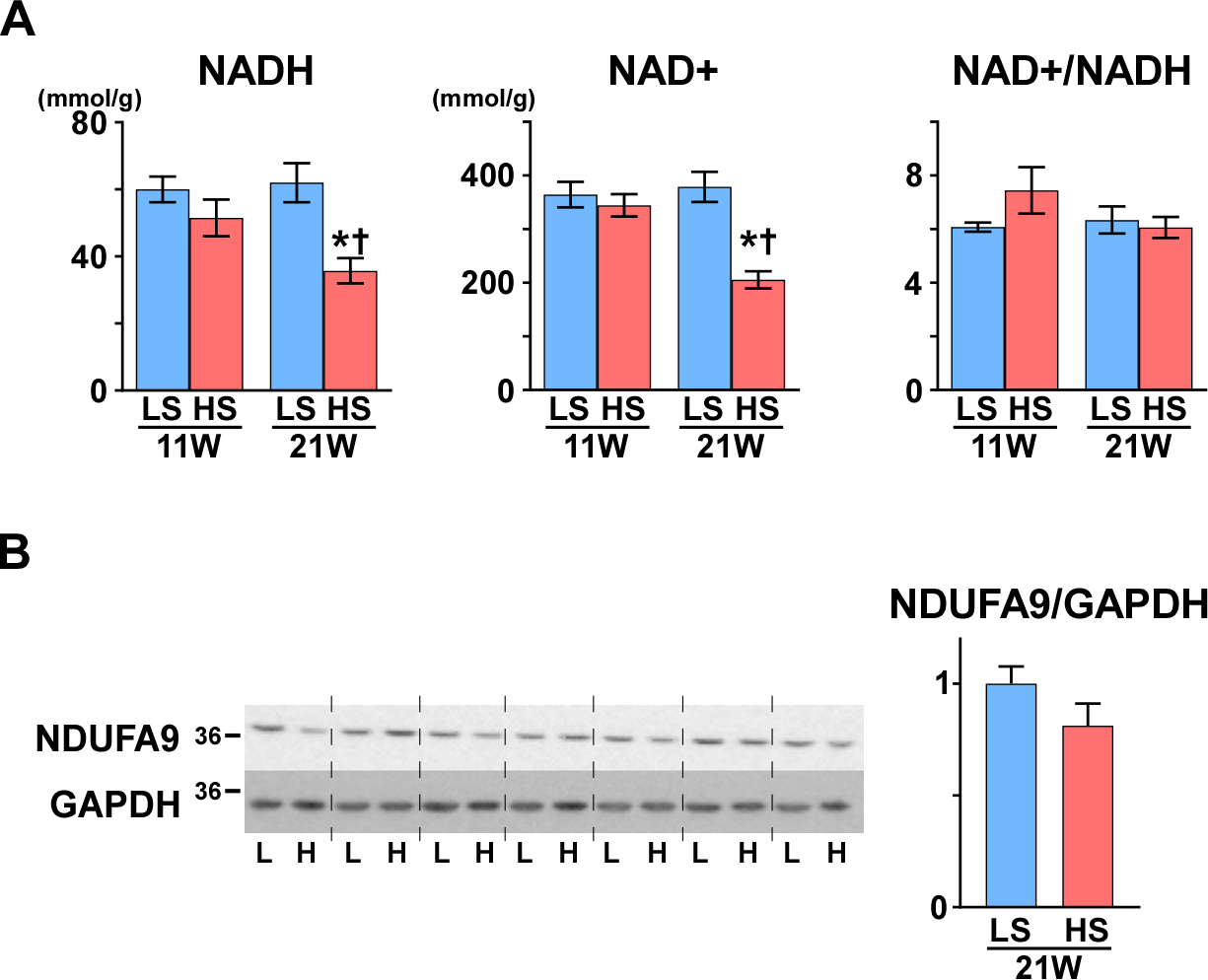
**
